# Management of Adverse Skeletal Effects Following Bariatric Surgery Procedures in People Living with Obesity

**DOI:** 10.1007/s11914-025-00902-9

**Published:** 2025-02-13

**Authors:** Léa Karam, Julien Paccou

**Affiliations:** 1 Department of Rheumatology, MABlab ULR 4490, CHU Lille, University Lille, 2, Avenue Oscar Lambret, 59037 Lille Cedex, France; 2 Saint-Joseph University, Beirut, Lebanon; 3 Rheumatology Department, Hotel-Dieu de France Hospital, Beirut, Lebanon

**Keywords:** Bariatric surgery, Weight loss, Vitamin D, Calcium intake, Physical activity, Bisphosphonates, Denosumab, Fractures, Bone mineral density

## Abstract

**Purpose:**

This review focuses on recent findi+ngs regarding the management of adverse skeletal effects following weight loss in people living with obesity (PwO). We summarize the guidelines provided by various societies for the prevention and treatment of osteoporosis resulting from bariatric surgery. Next, we discuss the use of traditional antiosteoporosis medications in this population.

**Recent Findings:**

Guidelines for preventing and treating osteoporosis resulting from bariatric surgery have been recently provided by various societies setting specific treatment criteria for postmenopausal women and men aged ≥ 50 years, based on the occurrence of fragility fractures and/or T-score thresholds. Several studies have highlighted the positive effects of lifestyle changes in preventing high-turnover bone loss; however, data on fracture outcomes are currently unavailable. It is generally accepted that following bariatric procedures, sufficient intake of calcium, vitamin D, and protein, along with regular exercise incorporating progressive, supervised resistance training, is crucial to counteract negative impacts on bone. Regarding the need for medications to combat osteoporosis, most societies recommend zoledronic acid as the preferred choice. This preference is due to the problems associated with oral bisphosphonates, including poor tolerance and absorption issues. Denosumab is typically considered the second choice when bisphosphonates are not suitable or well tolerated. Two randomized controlled studies have recently demonstrated the effectiveness and safety of zoledronic acid and denosumab in addressing high-turnover bone loss.

**Summary:**

Although guidelines exist for managing skeletal health before and after bariatric surgery, more research is required to validate these recommendations and the use of anti-osteoporosis medications.

**Supplementary Information:**

The online version contains supplementary material available at https://doi.org/10.1007/s11914-025-00902-9.

## Introduction

The increasing prevalence of obesity is a major global public health challenge. The prevalence of obesity has more than doubled since the 1990. In 2022, it was estimated that more than 890 million adults (16% of the adult population) are living with obesity [1]. The common health consequences of obesity include cardiovascular disease, hypertension, type 2 diabetes mellitus, metabolic disorders such as dyslipidemia and nonalcoholic fatty liver disease, certain cancers, and musculoskeletal disorders, such as osteoarthritis and low back pain [2].

Currently, the arsenal of obesity treatment includes lifestyle changes in combination with antiobesity medication (liraglutide, naltrexone/bupropion, orlistat, phentermine/topiramate, semaglutide, and tirzepatide) and bariatric surgery [3]. Although the screening and management of cardiometabolic complications of obesity are part of routine endocrine and bariatric clinical care, this is not the case for fracture risk. This review will address new developments in the prevention and treatment of adverse skeletal effects following bariatric surgery in people living with obesity (PwO).

## Search Strategy and Selection Criteria

References for the review were identified through PubMed searches for articles and reviews published between January 2022 and December 2024, although older references were used when appropriate. We used the search terms “fracture,” “osteoporosis,” “bariatric surgery” “gastric bypass,” “gastric banding,” “sleeve gastrectomy,” “biliopancreatic diversion” in combination with the terms “obesity” and “weight loss.” Articles revealed by these searches and relevant references cited in these articles were reviewed. Only articles published in English were included in the review.

## Skeletal Effects of Bariatric Surgery Procedures in PwO

Although bariatric surgery can result in significant weight loss and numerous health benefits [4], surgical weight loss is increasingly recognized to adversely affect bone metabolism [5]. The extent of high-turnover bone loss suggests severe bone impairment, and this phenomenon is larger than that observed in weight loss related to calorie restriction [5]. This is likely related, at least in part, to the magnitude of weight loss after bariatric surgery. The changes in bone turnover markers (BTMs) and bone mineral density (BMD) are similar to those found in calorie restriction: serum collagen type I cross-linked C telopeptide (CTX) levels increase to a greater extent than those of procollagen type I N-propeptide (PINP), and total hip BMD decreases to a greater extent than lumbar spine BMD [6, 7]. After Roux-en-Y gastric bypass (RYGB), PwO also experiences substantial deterioration in bone microarchitecture and strength, as assessed using high-resolution peripheral quantitative computed tomography (HR-pQCT) [8, 9].

**Fig. 1:**
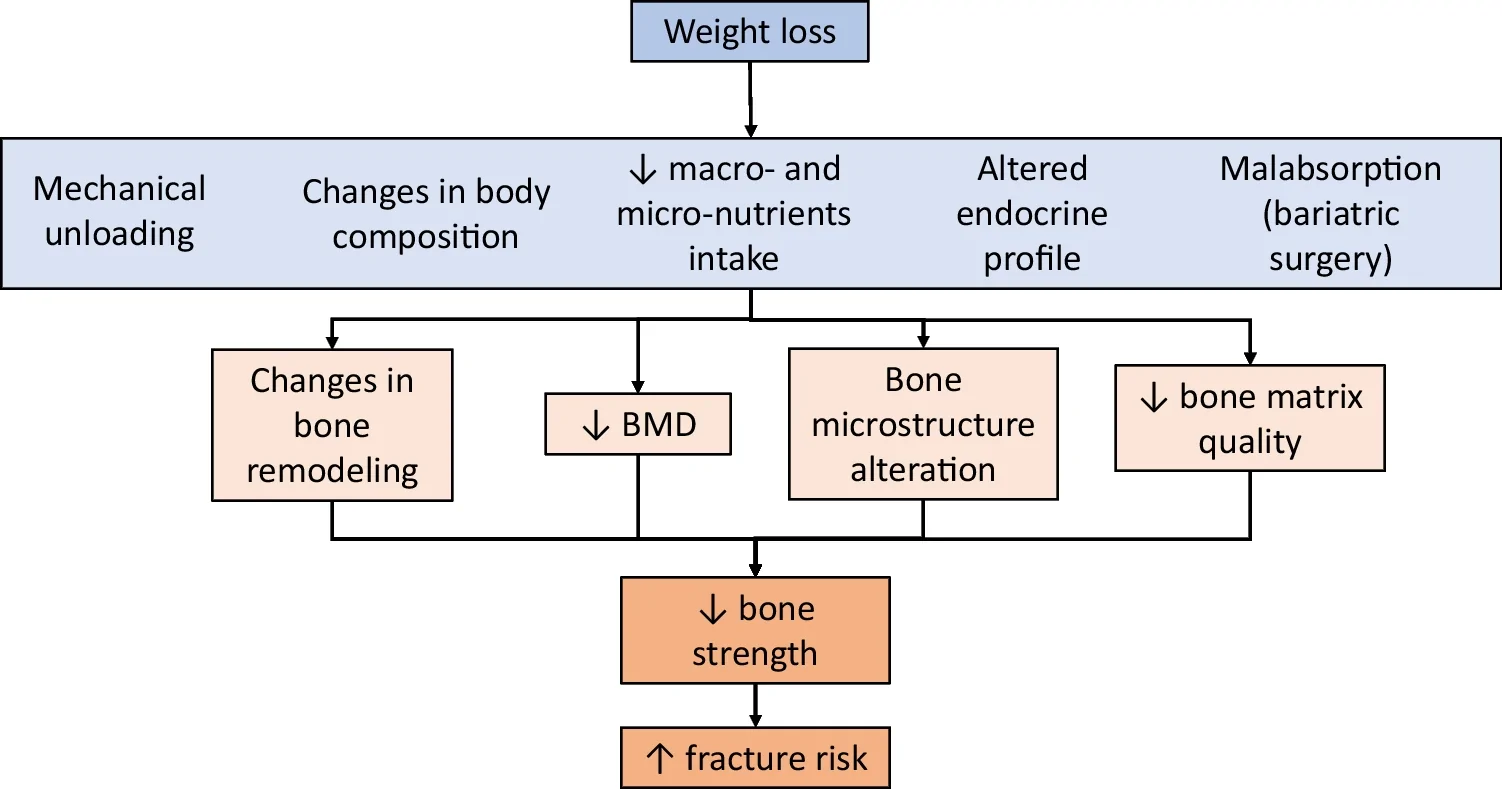
Mechanisms of bone loss associated with intentional weight loss, (adapted from Papageorgiou M & Biver E. Rev Med Suisse. 2023 Apr 19;19(823):756–760)

Undoubtedly, there is an association between surgical weight loss and a higher risk of fracture, which usually emerges in the 3rd year of follow-up [10, 11]. Although the underlying mechanisms are not fully understood, many factors are involved, including nutritional factors, mechanical unloading, loss of muscle mass, changes in the secretion of gut hormones and adipokines, and an increased risk of falls (Fig. 1) [5, 12]. Accumulating evidence suggests that RYGB is associated with a greater reduction in BMD, greater increase in BTMs, and higher risk of fragility fractures than sleeve gastrectomy (SG) [5, 10]. In addition, postmenopausal women are at highest risk for skeletal consequences than premenopausal women and men.

### Management of Adverse Skeletal Effects Following Bariatric Surgery

The American Society for Metabolic and Bariatric Surgery (ASMBS) released its initial position statement in 2015, with an update in 2020 [13]. Furthermore, guidelines for preventing and treating osteoporosis resulting from bariatric surgery were provided in 2022 by the European Calcified Tissue Society (ECTS) [14] and the joint efforts of the Osteoporosis Research and Information Group (GRIO) and French Rheumatology Society (SFR) [15]. A summary of these recommendations is provided in Table 1.

**Table 1:** Summary of the main guidelines on the prevention and treatment of osteoporosis secondary to bariatric surgery

	Who to assess?	How to assess?	Who to treat?	How to treat?
ASMBS (American Society for Metabolic and Bariatric Surgery) guidelines 2020 [13]	✓ All women aged 65 + and men aged 70 + ✓ Postmenopausal women and men above age 50–69, based on the risk factor profile ✓ Men aged 50 + who have had an adult age fracture	✓ Measurement of BMD by DXA ✓ Bone turnover markers (BTMs) can be considered	Not reported	General measures Calcium supplementation (1200–1500 mg/d after SG, AGB, and RYGB and 1800–2400 mg/d after BPD-DS) and attain a 25(OH) vitamin D concentration of at least 30 ng/mL. Increase of physical activity (aerobic and strength exercise) Anti-osteoporosis medication Not reported
ECTS (European Calcified Tissue Society) guidelines 2022 [14]	✓ Menopausal women and men ≥ 50 years ✓ Pre-menopausal women and men < 50 years • no routine BMD measurement • BMD measurement in patients at high risk of fracture*	✓ Measurement of BMD by DXA ✓ Spine radiographs or VFA ✓ Clinical Osteoporosis risk factors (CRFs) ✓ FRAX® ✓ Bone turnover markers (BTMs)	Menopausal women and men ≥ 50 years: • History of recent fragility fracture (> 40 years of age) • T-score ≤—2 at the lumbar spine and/or femur • FRAX® score with femoral neck BMD exceeding 20% for the 10-year MOF probability or exceeding 3% for hip fracture	General measures Treatment of vitamine D deficiency, optimization of total daily calcium and protein intakes as well as increase of physical activity (aerobic and strength exercise) Anti-osteoporosis medication Injectable bisphosphonates (zoledronate as first choice) Denosumab as second choice (contraindication or intolerance for bisphosphonates)
GRIO/SFR (Osteoporosis Research and Information Group – GRIO/ French Rheumatology Society – SFR) guidelines 2022 [15]	✓ Menopausal women and men ≥ 50 years ✓ Regardless of age, in the case of RYGB and biliopancreatic diversion ✓ Regardless of age, for patients at high risk of fracture*	✓ Measurement of BMD by DXA ✓ Vertebral imaging (if necessary) ✓ Clinical Osteoporosis risk factors (CRFs)	Menopausal women and men ≥ 50 years: • If previous history of severe fractures • If non-severe fracture and T-score ≤ −1 • If T-score ≤ −2 (in the absence of fractures) Who to refer? (to a specialist in bone diseases): Non-menopausal women and men < 50 years old • If previous history of bone fragility fractures • if Z-score ≤ −2 (in the absence of fractures)	General measures Normalise the intake of calcium (1000 mg/day after SG, and 1500 mg/day after RYGB) and protein (at least 60 g/day); attain a 25(OH) vitamin D concentration of at least 30 ng/mL; prevent the risk of falls and introduce a program of weight-bearing physical activity Anti-osteoporosis medication Zoledronic acid (cycle of 3 perfusions) No evidence supporting the benefit of using denosumab or oral bisphosphonates

*SG* sleeve gastrectomy, *RYGB* Roux-en-Y gastric bypass, *DBP* biliopancreatic diversion, *BMD* bone mineral density, *VFA* vertebral fracture assessment

^*^Patients at high risk of fracture are:

-those with a history of fragility fracture after the age of 40;

-those presenting comorbidities that are frequently associated with osteoporosis, i.e. certain endocrinopathies, neurological disorders with neurosensory impairment, hepatic cirrhosis, chronic obstructive pulmonary disease > stage 1, and chronic inflammatory diseases);

-those taking medications that are frequently associated with osteoporosis (corticosteroids, LH-RH antagonists, antiretroviral drugs, aromatase inhibitors, prolonged chemotherapy)

### Screening

For patients aged 50 years and older, including post-menopausal women, the evaluation should include clinical risk factors including a fracture history, alcohol consumption, and smoking habits, DXA testing of the lumbar spine and hip, spine radiographs or vertebral fracture assessment, measurement of BTMs, and biochemical analyses to identify secondary causes of osteoporosis. This assessment should ideally be performed before any bariatric surgery, regardless of the specific procedure [14, 15]. Some guidelines suggest that all patients undergoing RYGB and biliopancreatic diversion with duodenal switch (BPD/DS) should have their BMD and clinical risk factors evaluated due to the substantial bone loss and elevated fracture risk associated with these malabsorptive procedures [15]. Moreover, patients considered at high risk should undergo these evaluations, regardless of age. High-risk status is determined by either experiencing a fragility fracture after age 40 years, having comorbidities, or taking medications linked to osteoporosis (e.g., corticosteroids, aromatase inhibitors) [15].

A study by Blom-Høgestøl et al*.* investigated the occurrence of osteopenia, osteoporosis, and fragility fractures in 124 patients a decade after undergoing RYGB [16]. The study population had a mean age of 50.3 years (standard deviation (SD) 9.0), with 77% (*n* = 94) being female, of whom 44% (*n* = 41) were postmenopausal. Among the 59 participants who were postmenopausal women or men aged 50 years or older, osteopenia was found in 51% (*n* = 30) and osteoporosis in 27% (*n* = 16). This study did not provide information on the prevalence of BMD T-scores ≤  − 2. Fragility fractures were also common in this group. Nineteen percent (*n* = 11) of postmenopausal women and men aged 50 years or above reported having experienced a fragility fracture. DXA scans for vertebral fracture assessment revealed that 8% (*n* = 5) of the participants had at least one moderate or severe morphometric vertebral fracture [16].

A retrospective cohort study assessed the application of the ECTS guidelines in postmenopausal women and men aged ≥ 50 years who had undergone bariatric surgery [17]. This study, conducted from February 2019 to March 2022, included 170 patients (144 females; median age, 59 [55–63] years). The results showed that 33 patients qualified for anti-osteoporosis medication (AOM), indicating a prevalence of 19.6% [CI 95%: 13.9%–26.5%]. Most of these patients met the criteria based on a BMD T-score of −2 or lower (*n* = 25, 14.7% [CI 95%: 9.7%–20.9%]) and/or a recent history of fragility fractures (*n* = 12, 7.1% [CI 95%: 3.7%–12.0%]) [17]. This study was the first to evaluate the applicability and usefulness of the ECTS guidelines; however, the findings need to be confirmed to better determine the targeted population for evaluation and AOM in the context of surgical weight loss.

### Non-pharmacological Measures

Several studies have highlighted the positive effects of lifestyle changes in preventing bone loss; however, data on fracture outcomes are currently unavailable. It is generally accepted that following bariatric procedures, sufficient intake of calcium, vitamin D, and protein, along with regular exercise, is crucial to counteract the negative effects on the bone and muscle [5, 12].

#### Exercise Training

The BABS study, an interventional study, demonstrated a positive effect on BMD and BTMs through combined supplementation of vitamin D, calcium, and protein powder (35–60 g/day), coupled with aerobic exercise (Nordic walking, strength perseverance, and equipment training) [18] (Supplementary Table 1). The intervention group experienced a smaller decrease in total hip BMD (−3.9% vs. −9.9%, *p* < 0.001) and a smaller increase in BTMs (82.6% vs. 158.3%, *p* < 0.001 for CTX and 12.0% vs. 41.2%, *p* = 0.003 for PINP) than the control group. Three studies confirmed that a supervised exercise regimen consisting of weight-bearing and aerobic activities mitigated the reduction in BMD and BTMs typically observed following surgical weight loss [19–21]. In another study involving 154 patients, no treatment effect of exercise training was observed at 12 months on BMD, submitted to (single anastomosis, Roux-en-Y) gastric bypass, or SG. However, the exercise training duration was short [12 weeks], with only 1 session per week [22] (Supplementary Table 1).

Overall, studies have shown a lower decrease in BMD after an exercise training program, including both aerobic and resistance training. French guidelines suggest that aerobic and strength exercise programs alongside nutritional interventions may be particularly advantageous for PwO patients who have undergone bariatric surgery and could help reduce long-term bone loss [15].

#### Vitamin D Supplementation

Current recommendations exist for vitamin D supplementation before and after bariatric surgery [13–15, 23]. High-dose oral vitamin D3 supplementation (minimum 2,000 IU/day) is advised for PwO patients undergoing bariatric surgery, with intramuscular supplementation preferred over oral supplementation for those undergoing malabsorptive surgery [23]. A systematic review and meta-analysis provided data on vitamin D status and post-bariatric surgery vitamin D supplementation. The analysis included 39 studies with 5,296 patients [23]. Patients receiving high-dose oral vitamin D supplementation (≥ 2,000 IU/day, primarily the D3-formulation) showed lower rates of vitamin D insufficiency (25-hydroxyvitamin D (25(OH)D) < 30 ng/mL) and higher 25(OH)D levels post-surgery than those receiving low doses (< 2,000 IU/day), regardless of the type of procedure. For malabsorptive surgery, the prevalence of vitamin D insufficiency 6–24 months post-surgery was 43% versus 74% (*p* = 0.01), and 25(OH)D levels < 6 months postoperatively were 31 ng/mL versus 21 ng/mL (*p* = 0.03) [23].

#### Calcium Intake

Dietary calcium intake is reduced owing to calorie restriction induced by bariatric surgery. Furthermore, after RYGB and SG, the digestive absorption of calcium was reduced [24, 25]. A prospective observational cohort study examined the effect of SG on intestinal fractional calcium absorption (FCA) in 35 severely obese individuals aged 24–70 years [25]. The assessment was conducted six months after surgery, with participants maintaining adequate 25(OH)D levels and consuming the recommended calcium intake of approximately 1200 mg daily. The results showed a significant decrease in mean (SD) FCA from 31.4 ± 15.4% before surgery to 16.1 ± 12.3% after surgery (*p* < 0.01) [25]. This finding indicates that RYGB is not the sole bariatric procedure associated with reduced intestinal FCA [24]. Therefore, calcium intake (dietary ± supplementation) should be at least 1200 mg/day after SG and 1500 mg/day after RYGB. Dietary intake should be preferred, that is, dairy products (low-fat if needed) and calcium-rich mineral water, but since this does not always cover the 1200–1500 mg/day requirement, medical supplementation is often needed to attain these levels [14, 15]. Although there are insufficient data in the literature to support a preference for one form of calcium supplementation over another (calcium citrate, calcium carbonate, or other), in the absence of gastric acidity, calcium citrate may be better absorbed. If secondary hyperparathyroidism persists despite reaching an optimal 25(OH)D concentration between 75 nmol/L (30 ng/ml) and 150 nmol/L (60 ng/ml), a deficiency in calcium intake that induces a negative calcium balance should be considered [14, 15].

Interestingly, a study involving 20 postmenopausal women who had undergone RYGB an average of five years earlier was conducted to evaluate the impact of prebiotics on intestinal FCA [26]. This randomized, double-blind, placebo-controlled trial administered either prebiotics or placebo orally for a 2-month period. Unfortunately, the results showed no significant differences between the groups in terms of changes in FCA or calciotropic hormones [26].

In summary, all PWO who are candidates for bariatric surgery or have already undergone bariatric surgery should follow these measures: ensure adequate calcium and protein intake, achieve a serum 25(OH)D concentration ≥ 30 ng/mL, mitigate fall risks, and engage in an appropriate physical activity regimen (Table 2).

**Table 2:** Non-pharmacological prevention and treatment of bone health impairment in all Bariatric Surgery-treated patients

Rules to follow
-Adequate calcium intake (at least 1200 mg/day)
-Normalize the intake of protein (at least 60 g/day) – use of protein powder if needed
-Vitamin D ideally per day (at least 2000 units/day) throughout the year (attain a 25(OH) vitamin D concentration of 30–60 ng/mL)
-Prevention of falls
-Promote weight-bearing physical activity and progressive resistance training program
-No smoking
**-**Limited alcohol intake

### Pharmacological Intervention

Determining suitable standards for commencing AOM is crucial to address and prevent bone fragility caused by bariatric surgery. Various societies have differing views on the specific treatment criteria for postmenopausal women and men aged ≥ 50 years, based on the occurrence of fragility fractures and/or T-score thresholds [14, 15].

Most societies favor zoledronic acid (ZOL) as the primary option, because of issues with oral bisphosphonates intolerance and malabsorption [27]. Owing to safety reasons and the potential risk of anastomotic ulceration and direct gastric irritation, oral bisphosphonates should be avoided after bariatric surgery [27]. Denosumab is considered the secondary choice when bisphosphonates are contraindicated or not tolerated, owing to its associated risks, particularly the potential for rebound effects upon discontinuation [28, 29].

Information regarding the use of osteoanabolic agents for PwO after bariatric surgery is missing.

Nevertheless, the effectiveness of AOM in preventing bone loss following bariatric surgery remains partly unexplored, and there are currently no available data on fracture outcomes.

#### Bisphosphonates

Given the high-turnover bone loss state that occurs after bariatric surgery, it is logical to use inhibitors of bone resorption such as bisphosphonates.

#### Zoledronic Acid

A small-scale, open-label pilot study conducted by Liu et al*.* over 24 weeks examined the initial safety and effectiveness of ZOL in suppressing BTMs and preventing BMD loss after RYGB. A single administration of ZOL before RYGB seemed to temporarily reduce, but not completely halt, the increased bone turnover. Furthermore, while ZOL might help maintain trabecular volumetric BMD (vBMD) in the spine (as measured by quantitative computed tomography (QCT)), it appeared inadequate in preventing bone loss at the total hip (as assessed by DXA) [30].

These findings were confirmed in a recent randomized, double-blind, placebo-controlled study conducted at a single public hospital in Denmark (*the ZABAS study*). Patients undergoing RYGB or SG were randomly assigned (1:1) to receive ZOL (5 mg) or placebo (PBO) preoperatively [31]. The primary endpoint was the change in lumbar spine vBMD at 12 months postoperatively, assessed using QCT. The secondary outcomes included changes in the hip and femoral neck BMD, aBMD, and bone turnover markers (CTX and PINP). The 59 patients (mean (SD) age: 49·6 ± 6·6, BMI: 42·3 ± 5·3, female/male: 42/17) were randomized to either received ZOL (*n* = 31) or PBO (*n* = 28). The estimated mean treatment effects of ZOL for the spine and total hip were 7.2 mg/cm^3^ (95% CI 2.5, 11.9, *p* = 0.003) and 5.4 mg/cm^3^ (95% CI 1.7, 8.9, *p* = 0.003), respectively. Areal BMD loss at the lumbar spine was prevented in the ZOL group (+ 1.4%), whereas the PBO group experienced a decline of −4.1%. Additionally, bone loss at the total hip was blunted for ZOL compared to PBO (vBMD: −1.6% vs. −4.9%, *p* = 0.003). Both groups experienced significant aBMD loss at the total hip compared to baseline, although the loss was larger in the PBO group than in the ZOL group (−4.0% vs. −8.0%, *p* < 0.0001). The C-terminal telopeptide of type I collagen increased in both groups but was lower in ZOL than in PBO (+ 101.0% vs. + 172.0%, *p* = 0.01) [31] (Supplementary Table 2). Serious adverse reactions were not observed. Flu-like symptoms were significantly higher in the ZOL group than in the PBO group (*n* = 18 vs. *n* = 6; *p* = 0.02). The prevalence of hypocalcemia did not differ between the groups [31].

Another pilot randomized controlled trial of ZOL (versus PBO) to prevent bone loss following SG is ongoing at the University of Nebraska (NCT04279392). This protocol was published in 2021 [32].

#### Risedronate

This (*WE RISE)* pilot study investigated the effectiveness of 150 mg of monthly oral risedronate in preventing bone loss associated with SG [33]. A group of 24 patients (mean (SD) age, 56 ± 7 years; 83% female, 21% black) was randomly assigned to receive either risedronate or a PBO for six months. This study measured changes in aBMD using DXA. After six months, significant differences were observed between the groups in the femoral neck (risedronate: + 1.3% vs. placebo: −4.2%) and lumbar spine (risedronate: + 2.2% vs. placebo: −2.2%) (both *p* ≤ 0.02). However, risedronate was not effective in halving the 6-month aBMD loss at the total hip (−2.6% versus −4.4%). No difference in PINP was noted between the groups (risedronate: 4% versus PBO: 4%), but the increase in CTX was significantly lower in the risedronate group (+ 68%) than in the PBO group (+ 175%) (*p* < 0.001). Initial treatment effect estimates suggest that 6 months of risedronate use may be effective in reducing aBMD loss in the lumbar spine after SG [33] (Supplementary Table 2). No serious adverse events (ulceration or hypocalcemia) were encountered by patients in this study [33].

Further data from a sufficiently powered trial are necessary for confirmation (STRONG BONES study, NCT04922333) [34].

#### Denosumab

**Fig. 2:**
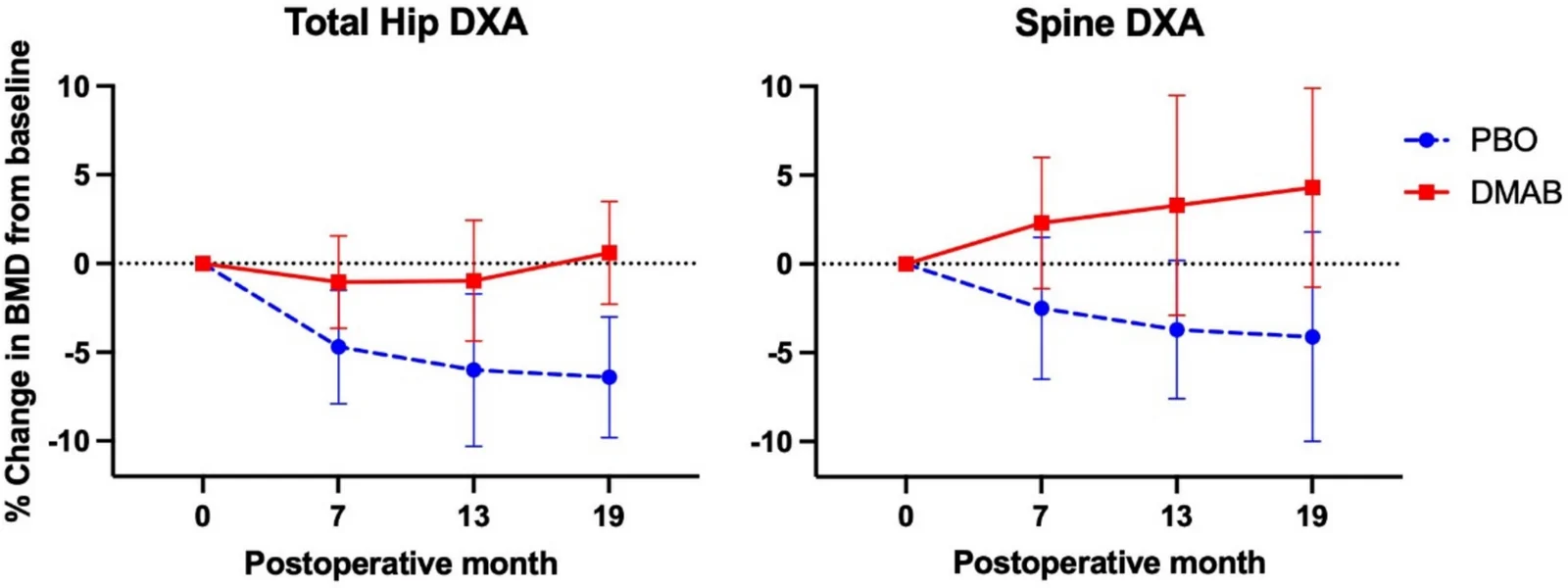
Effects of denosumab (DMAB) versus placebo (PBO) at 7, 13, and 10 months on total hip and lumbar spine areal bone mineral density assessed by dual energy X-ray in individuals undergoing Roux-en-Y gastric bypass or Sleeve Gastrectomy. Figure reproduced with permissions from Oxford University Publishing, Schafer [35]

Preliminary results on the efficacy and safety of denosumab (DMAB) for preventing bone loss in individuals undergoing RYGB or SG were reported during the last American Society for Bone and Mineral Research (ASBMR) 2024 Congress in Toronto [35]. In a 2-site, double-blind, randomized placebo-controlled trial of 36 postmenopausal obese women and men aged > 50 years undergoing RYGB or SG, participants were randomized 2:1 to receive either DMAB 60 mg or placebo (PBO) every 6 months for 18 months beginning one month after surgery. Calcium citrate and vitamin D supplements were titrated to achieve and maintain a total calcium intake of 1500 mg/day and 25(OH)D level > 30 ng/mL. At the hip and spine, aBMD was measured using DXA at baseline and 7, 13, and 19 months postoperatively, and vBMD was measured using QCT at 1 and 19 months postoperatively. All 36 randomized participants (DMAB, *n* = 24; PBO, *n* = 12) completed the full 19-month trial. The DMAB and PBO groups were similar in age (57 ± 7 years), sex (67% female), baseline BMI (44 ± 6 kg/m^2^), and surgery type (72% SG). The DMAB and PBO groups lost similar amounts of weight (−24.6 ± 15 kg over 19 months). Participants with DMAB had increased aBMD and vBMD at all sites compared to those in the placebo group (Fig. 2). For total hip aBMD (primary endpoint), the mean (95% CI) between-group difference over 19 months was + 7.0% (+ 4.7%, + 9.4%) (*p* < 0.001), with mean within-group changes −6.4% for PBO and + 0.6% for DMAB. For spine aBMD, the between-group difference was + 8.4% (+ 3.7%, + 13.1%) (*p* < 0.001), with mean changes −4.1% for PBO and + 4.3% for DMAB. The between-group differences in vBMD were + 9.0%, + 8.7%, and + 9.5% for the total hip, femoral neck, and spine, respectively (all *p* ≤ 0.03). Six DMAB participants each experienced asymptomatic grade 1 hypocalcemia once (corrected calcium 8.0–8.4 mg/dL), while no hypocalcemia occurred with PBO (*p* = 0.08). There were no hypocalcemia grades 2–4 and no study-related serious adverse events in either group. In conclusion, denosumab may be an effective and safe option to prevent loss of bone mass in postmenopausal women and men aged > 50 years undergoing bariatric surgery [35]. The publication of these results is awaited. Furthermore, infusion with ZOL was planned 6 months after the 3rd injection of denosumab to avoid a rebound effect [35].

A randomized, placebo-controlled trial evaluating the effects of denosumab in individuals undergoing bariatric surgery was presented at the 2024 American Society for Bone and Mineral Research (ASBMR) Annual Meeting. The study enrolled 36 adults (postmenopausal women and men older than 50 years) with obesity who underwent RYGB or SG. Participants were assigned in a 2:1 ratio to receive either denosumab (60 mg) or placebo at six-month intervals over an 18-month period, starting one month after surgery.

All participants received calcium and vitamin D supplementation adjusted to maintain a daily calcium intake of approximately 1500 mg and serum 25-hydroxyvitamin D levels above 30 ng/mL. Bone mineral density was assessed at multiple postoperative time points using DXA for areal measurements and quantitative computed tomography for volumetric analysis.

Baseline characteristics, including age, sex distribution, BMI, and type of surgical procedure, were comparable between the groups. Weight loss during the study period was also similar. However, individuals treated with denosumab demonstrated increases in both areal and volumetric BMD across the measured skeletal sites, whereas those receiving the placebo experienced declines. At the total hip, the difference between the groups over 19 months was approximately 7%, with additional significant differences observed at the spine (Fig. 2). Comparable improvements were observed in the volumetric measurements of the hip and spine.

Regarding safety, mild asymptomatic hypocalcemia was observed in a small number of participants receiving denosumab, whereas no such cases occurred in the placebo group. No severe hypocalcemia or treatment-related adverse events were reported.

Overall, these preliminary findings suggest that denosumab may represent a promising strategy for mitigating bone loss in older adults undergoing bariatric procedures, although a full peer-reviewed publication of the data is still pending.

## Conclusions

The field of skeletal health after bariatric surgery is evolving rapidly. This review summarizes our current knowledge on the prevention and treatment of adverse skeletal effects following bariatric surgery for PwO. Key concerns persist regarding who should undergo screening, how to conduct screening effectively, and when intervention is necessary. Clinicians should focus their attention on patients at high fracture risk, such as postmenopausal women and men older than 50 years. Before and after bariatric surgery, DXA should be used to measure BMD, and risk factors for osteoporosis should be assessed. Zoledronic acid as the first choice, together with appropriate vitamin D and calcium supplements, is preferred because of intolerance to oral bisphosphonate and malabsorption. Denosumab might be considered the secondary choice when ZOL is contraindicated or not tolerated. In patients at high risk of fracture, surgical interventions such as RYGB should be tailored to fracture risk, comorbidities, and desired weight loss.

Additional randomized controlled studies are needed to evaluate the effectiveness and safety of medications for osteoporosis to address high-turnover bone loss and manage osteoporosis in this population. Although guidelines exist for preserving skeletal health before and after bariatric surgery, more research is required to validate these recommendations [15, 16].

## Keys References

- Paccou J, Compston JE. Bone health in adults with obesity before and after interventions to promote weight loss. Lancet Diabetes Endocrinol. 2024 Oct;12(10):748–760.
  o Review of the literature on this topic written by 2 worldwide experts.
- Paccou J, Tsourdi E, Meier C, Palermo A, Pepe J, Body JJ, Zillikens MC. Bariatric surgery and skeletal health: A narrative review and position statement for management by the European Calcified Tissue Society (ECTS). Bone. 2022 Jan;154:116,236.
  o Review of the literature and expert opinion/European guidelines for management of bone health before and after bariatric surgery.
- Paccou J, Genser L, Lespessailles É, Bertin É, Javier RM, Duclos M, Joly AS, Boirie Y, Pattou F, Delarue J, Cortet B. French recommendations on the prevention and treatment of osteoporosis secondary to bariatric surgery. Joint Bone Spine. 2022 Nov;89(6):105,443.
  o Expert opinion/French guidelines for management of bone health before and after bariatric surgery.
- Giustina A, di Filippo L, Facciorusso A, Adler RA, Binkley N, Bollerslev J, Bouillon R, Casanueva FF, Cavestro GM, Chakhtoura M, Conte C, Donini LM, Ebeling PR, Fassio A, Frara S, Gagnon C, Latella G, Marcocci C, Mechanick JI, Minisola S, Rizzoli R, Santini F, Shaker JL, Sempos C, Ulivieri FM, Virtanen JK, Napoli N, Schafer AL, Bilezikian JP. Vitamin D status and supplementation before and after Bariatric Surgery: Recommendations based on a systematic review and meta-analysis. Rev Endocr Metab Disord. 2023 Dec;24(6):1011–1029.
  o Systematic review and meta-analysis of the literature and expert opinion/recommendations for management of vitamin D status and supplementation before and after Bariatric Surgery.
- Wu KC, Cao S, Weaver CM, King NJ, Patel S, Kim TY, Black DM, Kingman H, Shafer MM, Rogers SJ, Stewart L, Carter JT, Posselt AM, Schafer AL. Intestinal Calcium Absorption Decreases After Laparoscopic Sleeve Gastrectomy Despite Optimization of Vitamin D Status. J Clin Endocrinol Metab. 2023 Jan 17;108(2):351–360.
  o A clinical study to bring first evidence in humans that intestinal calcium absorption is decreased after sleeve gastrectomy.
- Gam S, Gram B, Juhl CB, Hermann AP, Hansen SG. Zoledronic Acid for prevention of bone and muscle loss after BAriatric Surgery (ZABAS)-a study protocol for a randomized controlled trial. Obesity. 2025. In Press.
  o This randomized controlled trial demonstrates that zoledronic acid seemed to temporarily reduce, but not completely halt, increased bone turnover and decreased bone mineral density.
- Schafer AL. Denosumab Increases Bone Mass in Post-menopausal Women and Older Men Undergoing Bariatric Surgery: A Randomized Controlled Trial. J Bone Miner Res. 2024. Annual Meeting.
  o This randomized controlled trial demonstrates that denosumab seemed to reduce the high turnover bone loss following bariatric surgery.

## Supplementary Information

Below is the link to the electronic supplementary material.

Supplementary file1 (DOCX 22 KB)

Supplementary file2 (DOCX 24 KB)

## Data Availability

No datasets were generated or analysed during the current study.
