# Correction: Management of Adverse Skeletal Effects Following Bariatric Surgery Procedures in People Living with Obesity

**DOI:** 10.1007/s11914-026-00981-2

**Published:** 2026-09-05

**Authors:** Léa Karam, Julien Paccou

**Affiliations:** 1 Department of Rheumatology, MABlab ULR 4490, CHU Lille, University Lille, 2, Avenue Oscar Lambret, 59037 Lille Cedex, France; 2 Saint-Joseph University, Beirut, Lebanon; 3 Rheumatology Department, Hotel-Dieu de France Hospital, Beirut, Lebanon

**Correction to**: Current Osteoporosis Reports

https://doi.org/10.1007/s11914-025-00902-9

The original published version of this article unfortunately contained an error.

In the published article the legend for figure 2 was as follows:

Fig. 2 Effects of denosumab (DMAB) versus placebo (PBO) at 7, 13, and 10 months on total hip and lumbar spine areal bone mineral density assessed by dual energy X-ray in individuals undergoing Roux-en-Y gastric bypass or Sleeve Gastrectomy

This should be corrected to:

Fig. 2 Effects of denosumab (DMAB) versus placebo (PBO) at 7, 13, and 10 months on total hip and lumbar spine areal bone mineral density assessed by dual energy X-ray in individuals undergoing Roux-en-Y gastric bypass or Sleeve Gastrectomy. Figure reproduced with permissions from Oxford University Publishing, Schafer [35]

Then an additional paragraph is added before the Conclusion section which states:

A randomized, placebo-controlled trial evaluating the effects of denosumab in individuals undergoing bariatric surgery was presented at the 2024 American Society for Bone and Mineral Research (ASBMR) Annual Meeting. The study enrolled 36 adults (postmenopausal women and men older than 50 years) with obesity who underwent RYGB or SG. Participants were assigned in a 2:1 ratio to receive either denosumab (60 mg) or placebo at six-month intervals over an 18-month period, starting one month after surgery.

All participants received calcium and vitamin D supplementation adjusted to maintain a daily calcium intake of approximately 1500 mg and serum 25-hydroxyvitamin D levels above 30 ng/mL. Bone mineral density was assessed at multiple postoperative time points using DXA for areal measurements and quantitative computed tomography for volumetric analysis.

Baseline characteristics, including age, sex distribution, BMI, and type of surgical procedure, were comparable between the groups. Weight loss during the study period was also similar. However, individuals treated with denosumab demonstrated increases in both areal and volumetric BMD across the measured skeletal sites, whereas those receiving the placebo experienced declines. At the total hip, the difference between the groups over 19 months was approximately 7%, with additional significant differences observed at the spine (Fig. 2). Comparable improvements were observed in the volumetric measurements of the hip and spine.

Regarding safety, mild asymptomatic hypocalcemia was observed in a small number of participants receiving denosumab, whereas no such cases occurred in the placebo group. No severe hypocalcemia or treatment-related adverse events were reported.

Overall, these preliminary findings suggest that denosumab may represent a promising strategy for mitigating bone loss in older adults undergoing bariatric procedures, although a full peer-reviewed publication of the data is still pending.

The original article has been corrected.

