# Supplementary material for: Management of Adverse Skeletal Effects Following Bariatric Surgery Procedures in People Living with Obesity
Source: Curr Osteoporos Rep. 2025 Feb 13;23(1):11. doi: 10.1007/s11914-025-00902-9 (PMC11825533; doi:10.1007/s11914-025-00902-9)
Supplement: Supplementary file 1 — Supplementary file1 (DOCX 22 KB) [file 11914_2025_902_MOESM1_ESM.docx]

**Supplementary Table 1: Characteristics and findings of trials assessing the effects of exercise on areal bone mineral density (g/cm²) using dual energy X-ray (DXA)**

| Reference  Follow-up duration | Study design  Surgery type | Population | Intervention | Results |
| --- | --- | --- | --- | --- |
| Muschitz et al.^18^  24 months | RCT  RYGB: 61%  SG: 39% | Exercise group: N = 110  - Age: 41.0 (34;0; 45.0) years  - BMI: 44.3 (41.1; 47.9) kg/m²  - Female: 60%  Control group: N = 110  - Age: 40.0 (35.0; 45.8) years  - BMI: 44.2 (40.7; 47.7) kg/m²  - Female: 56% | Post-operative intervention  - Program duration: 24 months  - Start: 2 weeks after surgery  - Aerobic + resistance training + protein, calcium and vit. D supplementation  - 5 sessions/week of aerobic training (45 min, intensity not reported) + 2 sessions/week of resistance training (30 min, intensity not reported)  - Supervision: none  Control group: usual care | **Baseline → 24 months**  Intervention effect  Estimate (SE); p-value  TH: -3.9% (-4.4, 1.0) versus -9.9%  (-12.4, -7.7); p=0.014  FN: -3.2% (-4.1, -0.9) versus -6.7% (-10.5, -6.4); p=0.011  LS: -1.2% (-1.7, -0.7) versus -7.9%  (-10.0, -7.1); p<0.001 |
| Murai et al.^19^  9 months | RCT  RYGB: 100% | Exercise group: N = 31  - Age: 40.0 (7.8) years  - BMI: 49.8 (7.0) kg/m²  - Female: 100%  Control group: N = 32  - Age: 42.1 (8.2) years  - BMI: 48.5 (8.1) kg/m²  - Female: 100% | Postoperative intervention  - Program duration: 6 months  - Start: 3 months after surgery  - Aerobic + resistance training  - 2 sessions/week: 30–60 min of moderate-intensity aerobic exercise + 3 sets of 8–12 rep on 7 resistance exercises  - Supervision: total (staff not reported)  Control group: usual care | **Baseline → 9 months**  Exercise group  TH: -5.00% (2.80%)  FN: -4.41% (3.30%)  LS: -0.52% (3.73%)  Control group  TH: -7.26% (2.90%)  FN: -7.33% (3.70%)  LS: -1.43% (5.20%)  Intervention effect  Estimated mean difference (EMD); p-value  TH: -2.26%; p=0.009  FN: -2.91%; p=0.007  LS: NA; p=0.497 |
| Campanha-Versiani et al.^20^  12 months | Non-RCT  RYGB: 100% | Exercise group: N = 18  - Age: 37.2 (9.3) years  - BMI: 42.5 (4.0) kg/m²  - Female: 83%  Control group: N = 19  - Age: 37.0 (10.8) years  - BMI: 41.7 (4.6) kg/m²  - Female: 83% | Postoperative intervention  - Program duration: 9 months  - Start: 3 months after surgery  - Aerobic + resistance training  - 2 sessions/week: 1–3 series of 10–12 rep on 8 resistance exercises + 25 min of aerobic exercise (70–80% RHR)  - Supervision: total (staff not reported)  Control group: usual care | **Baseline → 12 months**  Exercise group  TH: 1.29 (0.17) → 1.26 (0.18)  LS: 1.21 (0.13) → 1.19 (0.13)  Control group  TH: 1.31 (0.14) → 1.22 (0.14)*  LS: 1.22 (0.14) → 1.13 (0.14)*  *p < 0.05 compared with time 0 (paired Student’s t test)  Intervention effect  Delta values; p-value of delta values  TH: −0.04 versus −0.09**; p<0.001  LS: −0.02 versus −0.09**; p=0.013  **p < 0.01 compared with time 0 (paired Student’s t test) |
| Diniz-Sousa et al.^21^  12 months | RCT  RYGB: 69%  SG: 31% | Exercise group: N = 41  - Age: 41.6 (10.5) years  - BMI: 44.2 (6.8) kg/m²  - Female: 83%  Control group: N = 20  - Age: 46.5 (8.5) years  - BMI: 46.1 (4.2) kg/m²  - Female: 80% | Post-operative intervention  - Program duration: 11 months  - Start: 1 month after surgery  - Aerobic + resistance training  - 3 sessions per week: 75 minutes each including resistance training (35 minutes)  - Supervision: total (exercise trainer)  Control group: usual care | **Baseline → 12 months**  Intervention effect  between-group differences; p-value  TH: −0.009 g/cm² ; p=0.431  [95% CI −0.031, 0.013]  FN: +0.021 g/cm² ; p=0.109  [95% CI −0.004, 0.046]  LS: +0.024 g/cm² ; p=0.015  [95% CI 0.004, 0.044] |
| Jassil et al.^22^  12 months | RCT  OAGB; 16%  RYGB: 29%  SG: 55% | Exercise group: N = 79  - Age: 44.8 (10.8) years  - BMI: 42.1 (5.8) kg/m²  - Female: 49%  Control group: N = 74  - Age: 43.6 (10.5) years  - BMI: 42.7 (5.7) kg/m²  - Female: 51% | Post-operative intervention  - Program duration: 12 weeks  - Start: 3 months after surgery  - Aerobic + resistance training  - 1 session per week + 17 nutritional-behavioural tele-counselling sessions for 12 months  - Supervision: total (exercise therapists)  Control group: usual care | **Baseline → 12 months**  Exercise group  TH: 1.173 (0.141) → 1.085 (0.143)  FN: 0.950 (0.160) → 0.895 (0.164)  LS: 1.158 (0.159) → 1.122 (0.172)  Control group  TH: 1.133 (0.107) → 1.032 (0.116)  FN: 0.907 (0.120) → 0.839 (0.113)  LS: 1.108 (0.148) → 1.092 (0.112)  Intervention effect  Estimate (SE); p-value  TH: 0.003 (0.004); p=0.48  FN: 0.003 (0.005); p=0.51  LS: -0.003 (0.004); p=0.40 |
|  |  |  |  |  |

RHR: reserve heart rate;
