# Supplementary material for: Management of Adverse Skeletal Effects Following Bariatric Surgery Procedures in People Living with Obesity
Source: Curr Osteoporos Rep. 2025 Feb 13;23(1):11. doi: 10.1007/s11914-025-00902-9 (PMC11825533; doi:10.1007/s11914-025-00902-9)
Supplement: Supplementary file 2 — Supplementary file2 (DOCX 24 KB) [file 11914_2025_902_MOESM2_ESM.docx]

**Supplementary Table 2: Pharmacological prevention and treatment of bone health impairment in all Bariatric Surgery-treated patients**

| Study | Population | Obesity classification  Criteria | Type of bone outcomes | Results |
| --- | --- | --- | --- | --- |
| Gam et al., **RCT 1:1**  *Obesity. 2025 ^31^*   - Intervention (before surgery): ZOL 5mg once versus PBO - Duration: 12 months - Calcium and vitamin D suppl: Calcium: 800 mg daily   Vitamin D: 38 μg daily   - Protein intake: NA - Weight loss: ~25% (~−31 kg) - Physical activity: NA | n=**59**,  men (28.8%) and women (71.2%),  aged >35 y  Aged 49.6 ± 6.6 y  Procedures: RYGB (68%) and SG (32%)  Menopausal status: NA  T2DM: NA | Obese ≥ 35 kg/m²  BMI: 42.3 ± 5.3 kg/m²  BMD-criteria: No  Fractures: NA | QCT: vBMD LS, FN, and TH  DXA: aBMD LS, FN, and TH  BTMs: CTX and PINP | **Baseline → 12 months**  Intervention effect (ZOL vs. PBO)  **QCT**  LS (+0·8% vs. -4.1%); p=0.003  FN (-1.2% vs. -4.1%); p=0.056  TH (-1.6% vs. -4.9%); p=0.003  **DXA**  LS (+1.4% vs. -3.3%); p=0.003  FN (-1.4% vs. -6.3%); p=0.015  TH (-4% vs. -8%); p=0.002  **BTMs**  CTX (+101% vs. +172%); p=0.011  PINP (+12.8% vs. +76.5%); p<0.0001 |
| Beavers et al. **RCT**  *Clinical Obesity. 2021 ^33^*   - Intervention (3-7 days before surgery): oral RIS 150 mg monthly (n=11) versus PBO (n=13) - Duration: 6 months - Calcium and vitamin D intakes:   Total calcium intake of 1200-1500 mg/day  At least 3000 IU/d Vitamin D to obtain 25OHD levels > 30 ng/mL   - Protein intake: NA - Weight loss: ~-17 kg (~15%) - Physical activity: exercice recommendations including daily walking and strength training | n=**24**,  men (17%) and women (83% including 63% of postmenopausal women),  Age 40-79  Aged 56 ± 7 y  Procedures: SG (100%)  T2DM: NA | Obese ≥ 35 kg/m²  BMI: 44.7 ± 6.3 kg/m²  BMD-osteoporosis: 0  BMD-Osteopenia: 3  Fractures: 11 | DXA: aBMD LS and TH  BTMs: CTX and PINP | **Baseline → 6 months**  Intervention effect (RIS vs. PBO)  **DXA**  LS (2.2% vs. -2.2%); p=0.013  FN (1.3% vs. -4.2%); p=0.016  TH (-2.6% vs. -4.4%); p=0163  **BTMs**  CTX (+68% vs. +175%); p<0.001  PINP (-4% vs. -4%); p=0.98 |

ZOL: zoledronic acid, PBO: Placebo, RYGB: Roux-en-Y Gastric Bypass, SG: Sleeve Gastrectomy
